# The Colloidal State of Matter

**Published:** 1928

**Authors:** F. Francis

**Affiliations:** Professor of Chemistry, University of Bristol


### THE COLLOIDAL STATE OF MATTER.

BY

F. FRANCIS, D.Sc.,

*Professor of Chemistry, University of Bristol.*

---

In 1861 Graham found that substances which dissolved in water could be classified into two groups. Bodies like sugar, salt and urea in solution rapidly diffuse through parchment, and these solutions deposit crystals on evaporation; this group was termed Crystalloids. On the other hand, glue, dextrin, gelatin in solution either diffuse very slowly or not at all, and as a general rule such solutions do not crystallise on evaporation; this group was termed Colloids. It is preferable to speak of a substance as being in the "colloidal state" rather than allude to it as a "colloid," for in all probability all substances can be caused to assume this state.

In true solutions the dissolved substance is either in the molecular or ionic condition, and such solutions are looked upon as homogeneous mixtures; colloidal solutions, however, are heterogeneous. These latter solutions may be clear and transparent to the naked eye, and no microscope—used in the normal way—reveals the solid phase, but if a beam of light is passed through them, the beam viewed at right angles to its path, the track of that beam appears turbid; in true solutions the track is invisible. The light diffracted is often polarised, indicating that the particles must have dimensions comparable to the wave length of the illuminating beam. If this beam is examined with a microscope placed at right angles to the track—*i.e.* the "ultra-microscope"—the diffraction images of the separate particles are rendered visible. The particles themselves are invisible; it is the light reflected from them only that is seen. If the liquid is optically clear (a pure solvent, or solutions of crystalloids) the field of view is dark; if matter is present in the colloidal state, minute points of light are seen against the dark background. These are in more or less rapid motion. If a colloidal solution is being examined, the field of view appears to be filled with brilliantly-lit particles; "like a swarm of dancing gnats in a sunbeam, they spring, hop, dance and fly apart so rapidly that the eye can hardly follow them."

The diameter of a blood corpuscle is about one eight-thousandth of a millimetre, a "micron" $\mu$ = ·001 mm., hence a corpuscle is about 8 $\mu$. Most micrococci are about 1 $\mu$. Particles of colloidal gold are about seven to fifteen-millionths of a millimetre; as 1 $\mu\mu$ = ·000001 mm., such colloidal material lies between 7 and 15 $\mu\mu$. A molecule of chloroform is about 0·8 $\mu\mu$, and a molecule of hydrogen 0·1 $\mu\mu$.

The characteristic properties of material in the colloidal state are largely due to the immense development of surface. A small lead shot converted into this state develops a surface many million times that of lead in the form of shot.

The motion of matter in the colloidal state is due to the ceaseless movement of the molecules in the interior part of all liquids. The particles behave as if they were true gas molecules with kinetic energy.

The colloidal particles are electrically charged, and many of their properties, especially their stability, depend on the nature of this charge. Silver chloride, or lead selenide, for instance, are negatively charged, and the solution bathing them positively. On the other hand, lead prepared by Bredig's method, or ferric hydrate, is positively, and the liquid in which they exist negatively, charged.

Substances in the colloidal condition can be divided into two groups. (1) *Suspensoids* (or suspensions or sols), such, for instance, as lead, silver, selenium, iodine, lead selenide, silver bromide; all are either positively or negatively charged in solution in water, and are precipitated on discharging, negatively charged colloids by positively charged ions, or positively charged particles by negatively charged ions. During this discharging process the precipitated colloid carries with it a part of the precipitating ion, a phenomenon known as adsorption and highly characteristic of colloidal phenomena. (2) *Emulsoids* (or jellies or gels), such as gelatin, gum arabic, glue, starch, dextrin, etc., etc. These also carry electrical charges, are heavily hydrated, but are not precipitated by dilute solutions of electrolytes. Heat, however, or the addition of concentrated solution of salt or other solvents, acting upon the hydrated colloidal particles, effects coagulation in such cases.

The difference between suspensoids and emulsoids is a matter of degree; probably the latter occupy a position intermediate between suspensoids and true solutions. Under the ultra-microscope emulsoids show a general diffuse light. There is not in these solutions the same optical contrast between the two phases as there is in the case of suspensoids. Emulsoids, particularly those not sensitive to precipitation by electrolytes, increase to a surprising extent the stability of suspensoids towards such electrically charged ions. Consequently, colloidal solutions of lead selenide, for instance, may be stabilised by the addition of solutions of gum arabic ; in such cases the latter substance is termed the *protective colloid.*

Within recent years an ever-increasing number of substances has been obtained in the colloidal state by methods which cannot be described here. Suspensoids of various elements, such as lead, selenium, iodine, etc., and compounds such as lead selenide, etc., have been used in medicine, but biologically emulsoids are by far the more important group. The latter contain starch, glycogen, proteins, lecithin, enzymes, etc., etc., and even their enumeration is sufficient to show that all the phenomena of life are connected with processes that take place only in colloidal systems.

